# Risk of bleeding in transbronchial biopsies in patients with pulmonary hypertension

**DOI:** 10.1038/s41598-026-42775-7

**Published:** 2026-03-24

**Authors:** Juan Ricardo Lutz, Javier Leonardo Galindo, Vanessa Barbosa

**Affiliations:** 1 Department of Pneumology, Hospital Universitario Mayor Méderi, Bogotá, D.C, Colombia; 2 School of Medicine, Universidad del Rosario, Bogotá, D.C, Colombia

**Keywords:** Bronchoscopy, Pulmonary hypertension, Hemorrhage, Cohort studies, Colombia, Risk factors, Respiratory tract diseases

## Abstract

This study aimed to identify key risk factors associated with bleeding after transbronchial biopsies in patients living in Bogotá, Colombia (2,640 m above sea level). A retrospective cohort study was conducted in adult patients who underwent bronchoscopy with transbronchial biopsies. Postbronchoscopy bleeding was classified according to the British Thoracic Society criteria. Logistic regression analyses were performed to investigate the relationships between the variables and postbronchoscopy bleeding. Among 208 participants (54.3% male, median age 69 years, IQR 56–77), bleeding occurred in 78 procedures (37.5%), most of which were mild (76.9%). A moderate to high probability of pulmonary hypertension on echocardiography was found in 31.2% of patients; a high probability of pulmonary hypertension on computed tomography scan was found in 66.8% of patients. No significant associations were found between bleeding risk and a moderate to high probability of pulmonary hypertension on echocardiography (OR 0.79, 95% CI 0.28–2.15; *p* = 0.896) or a high probability on computed tomography (OR 0.66; 95% CI 0.29–1.49; *p* = 0.848). Chronic obstructive pulmonary disease was associated with postbronchoscopy bleeding (OR 2.79, 95% CI 1.26–6.19; *p* = 0.012). The results support that the probability of pulmonary hypertension on echocardiography or computed tomography is not associated with postbronchoscopy bleeding but suggest that chronic obstructive pulmonary disease is a relevant factor to consider.

## Introduction

Bronchoscopy is a diagnostic and therapeutic procedure used to examine the airways and lungs in a minimally invasive manner^1^. Although it is usually safe, some complications may occur, especially in older patients with comorbidities^2,3^. Bleeding is most common when transbronchial biopsy (TBB) is performed, and the incidence of significant bleeding is approximately 1–5% of procedures^4–7^. It rarely leads to hemodynamic instability but can worsen hypoxemia and cause respiratory failure^7^. Risk factors associated with bleeding include the type of biopsy (transbronchial, endobronchial, or cryobiopsy), lesion diameter (≥ 30 mm), lesion location (hilar in the upper or middle lobe), additional biopsies, older patient age (≥ 75 years), male sex, high body mass index (≥ 25 kg/m2), smoking, renal failure, thrombocytopenia, immunosuppression, lung transplant recipients and the use of mechanical ventilation^1,8^. Anticoagulant and antiplatelet therapy also increases the risk of major bleeding in patients undergoing TBB; severe bleeding (> 100 mL) is common with the use of clopidogrel (89%) and is even higher with the use of combination therapy with clopidogrel and aspirin (100%)^1,9,10^.

Bleeding usually originates from bronchial arteries, where blood pressure is high, and rarely originates from the pulmonary vascular system^11,12^. Nevertheless, pulmonary hypertension has been mentioned as a contraindication for TBB, as it could theoretically increase capillary pressure and vasodilate submucosal bronchial veins^8,13^. Furthermore, it may cause hemodynamic changes that impact right ventricular function, resulting in cardiac arrhythmias and ischemia^14^. Inhabitants at high altitudes are chronically exposed to lower inspired oxygen partial pressure, which promotes physiological changes such as erythrocytosis and increased pulmonary pressure^15,16^. Patients who undergo bronchoscopy at high altitudes may be at increased risk of bleeding if they have pulmonary hypertension; to our knowledge, no studies have assessed the risk of bleeding in this population^17^. To address this hypothesis, we used real-world hospital-based retrospective research data aimed at identifying prognostic factors for bleeding due to TBB, including echocardiographic and tomographic probabilities of pulmonary hypertension, in patients living in Bogotá, Colombia.

## Results

### Study population

We included 208 patients (54.3% male) who underwent TBB (Table 1). The participants ranged in age from 17 to 91 years; the median age was 69 years (IQR 56–77). Postbronchoscopy bleeding was reported in 78 procedures (37.5%); most cases were mild (*n* = 60, 76.9%), with few moderate cases (*n* = 17, 21.8%). Only one case was categorized as severe and required mechanical ventilation and intensive care unit (ICU) admission. No deaths were recorded.

**Table 1:** Characteristics of the study population according to the occurrence of bleeding.

	Bleeding (n= 78)	Nonbleeding (n= 130)	*p*-value
Age, years	71 (56.3-77)	68 (56.3-76)	0.56
Sex, male (%)	42 (53.8)	71 (54.6)	0.91
Comorbidities			
Chronic obstructive pulmonary disease	20 (12.3)	16 (12.3)	0.01
Heart failure	11 (14.1)	18 (13.8)	0.96
Chronic kidney disease	13 (16.7)	8 (6.2)	0.02
Asthma	2 (2.6)	3 (2.3)	1
Interstitial lung disease	1 (1.3)	3 (2.3)	1
Pulmonary hypertension (diagnosed by right heart catheterization)	1 (1.3)	3 (2.3)	1
Cirrhosis	1 (1.3)	0 (0)	-
Thrombocytopenia	4 (5.1)	8 (6.1)	0.86
Indications for transbronchial biopsy (%)			
Lung mass	31 (39.7)	44 (33.8)	0.39
Miliary micronodules	13 (16.7)	23 (17.7)	0.85
Lung nodules	15 (19.2)	18 (13.8)	0.3
Organizing pneumonia	8 (10.3)	12 (9.2)	0.81
Perilymphatic micronodules or interlobular septal thickening	1 (1.3)	10 (7.7)	0.09
Cavitation	4 (5.1)	7 (5.4)	1.00
Persistent lung consolidation	4 (5.1)	6 (4.6)	1.00
Centrilobular micronodules	2 (2.6)	3 (2.3)	1.00
Other	0 (0)	7 (5.4)	-
Number of biopsies taken during bronchoscopy			
≤5	28 (35.9)	27 (20.8)	0.5
>5	31 (39.7)	23 (17.7)	
Missing values	19 (24.4)	80 (61.5)	
Echocardiographic findings			
sPAP:			0.58
<40 mmHg	61 (78.2)	100 (76.9)	
40-60 mmHg	12 (15.4)	25 (19.2)	
>60 mmHg	5 (6.4)	5 (3.8)	
Pulmonary hypertension probability:			0.99
Low	54 (69.2)	89 (68.5)	
Moderate	13 (16.7)	22 (16.9)	
High	11 (14.1)	19 (14.6)	
CT scan of the chest findings			
Pulmonary artery diameter	29.1 ± 0.5	29.1 ± 0.4	0.93
Pulmonary artery enlargement (>29 mm in men/>27 mm in women)	44 (56.4)	70 (53.8)	0.83
Pulmonary artery to aorta diameter ratio >0.9	34 (43.6)	67 (51.5)	0.33
Pulmonary hypertension probability:			0.85
Low	27 (34.6)	42 (32.3)	
High	51 (65.4)	88 (67.7)	

Data are presented as the mean ± standard deviation, median(interquartile range) or n (%). CT: computed tomography; sPAP: systolic pulmonary arterial pressure.

The most common indication for bronchoscopy was pulmonary masses (*n* = 75, 36.1%), followed by miliary micronodules (*n* = 36, 17.3%) and pulmonary nodules (*n* = 33, 15.9%). Other common indications were organizing pneumonia, lymphangitic carcinomatosis, and cavities. COPD (*n* = 36, 17.3%) and heart failure (*n* = 29, 13.4%) were the most common underlying comorbidities. At the time of bronchoscopy, 23 patients were receiving antiplatelet therapy with aspirin (*n* = 21, 10.1%) or combination therapy with clopidogrel and aspirin (*n* = 2, 1.0%). Only five patients on antiplatelet therapy experienced bleeding; three patients taking aspirin had mild bleeding, and two patients on combination therapy had moderate bleeding.

The probability of pulmonary hypertension on echocardiography was moderate to high in 31.2% of patients; systolic pulmonary arterial pressure (sPAP) > 40 mm was found in 22.6% of patients. The probability of pulmonary hypertension according to the computed tomography (CT) scan of the chest was high in 66.8%; 54.8% had increased pulmonary artery diameter, and 48.6% had an increased pulmonary artery to aorta diameter ratio. The agreement between CT and echocardiography for a high probability of pulmonary hypertension was slight (Cohen’s kappa 0.16, 95% CI 0.06–0.26; *p* = 0.001).

### Analysis of prognostic factors for postbronchoscopy bleeding

**Fig. 1:**
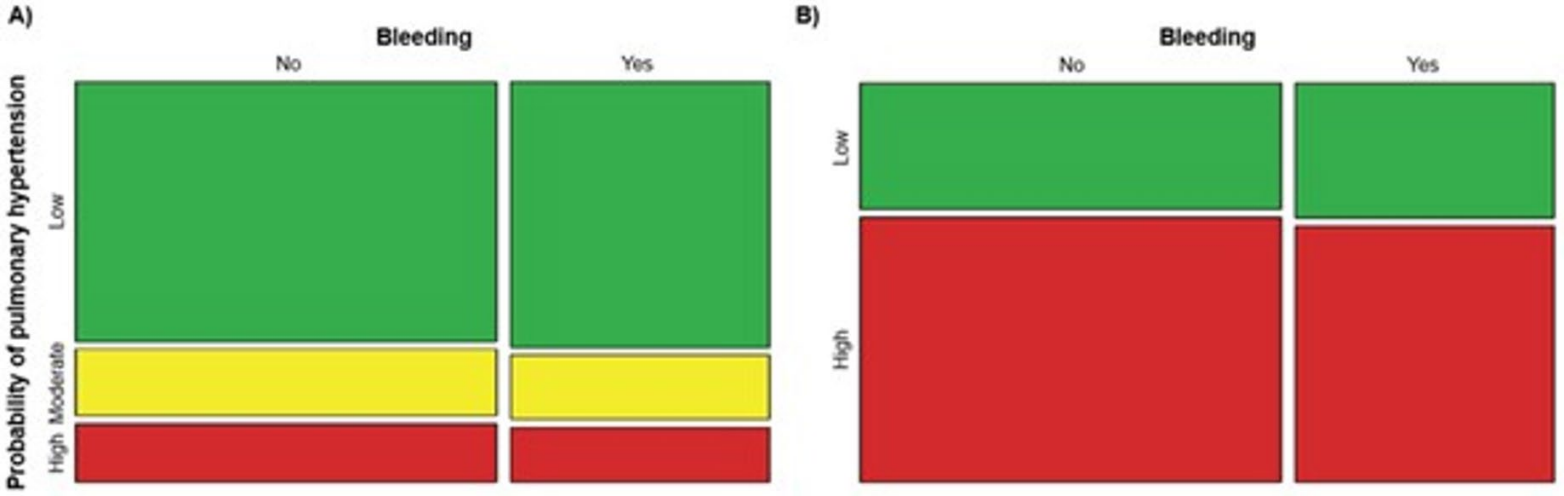
Bleeding due to TBB according to echocardiographic (**A**) and CT (**B**) probabilities of pulmonary hypertension.

**Fig. 2:**
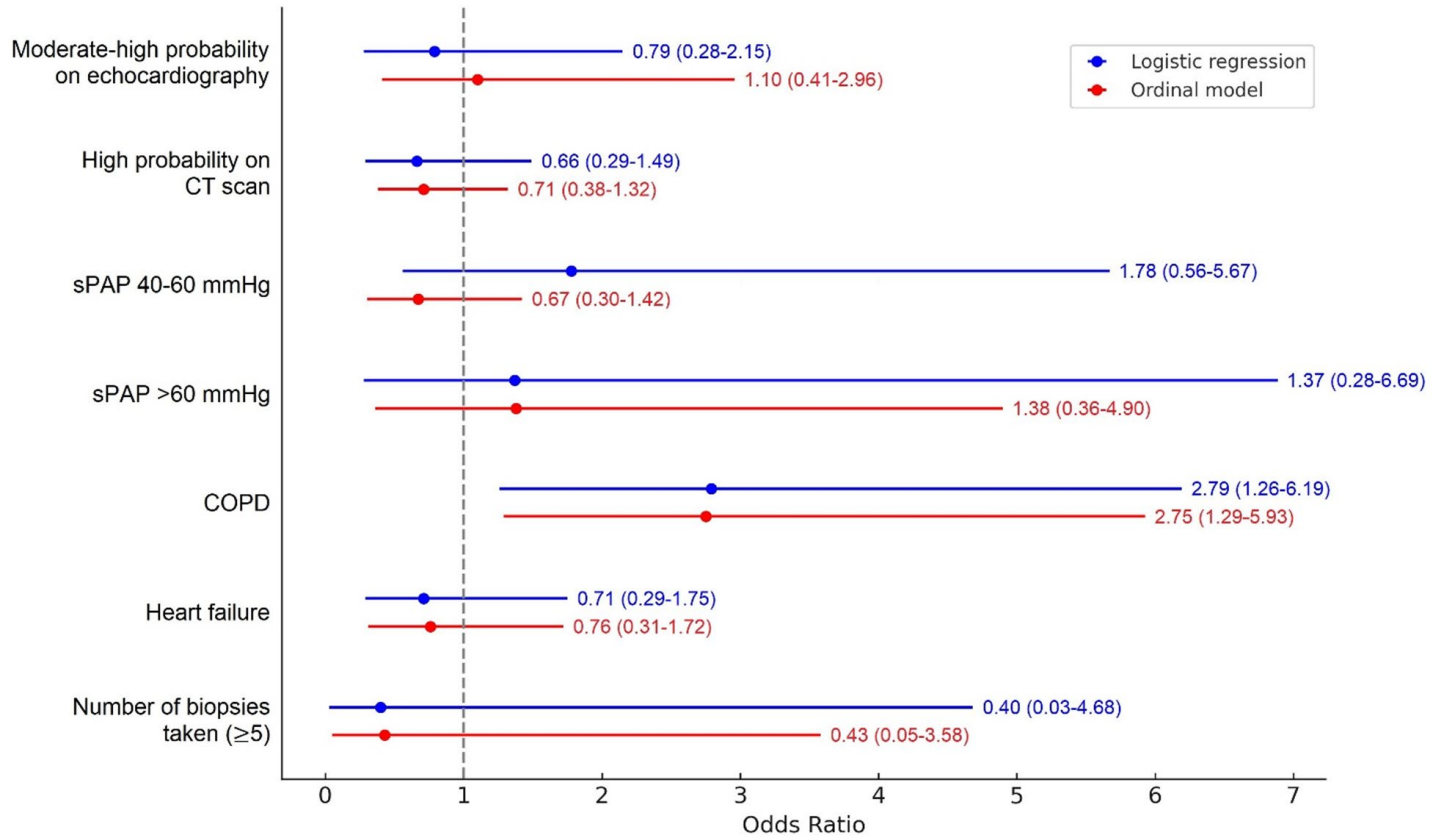
Forest plot for the risk factors for postbronchoscopy bleeding in logistic and ordinal models. *COPD* Chronic obstructive pulmonary disease, *CT* computed tomography, *sPAP* systolic pulmonary arterial pressure.

The mosaic plot comparing bleeding cases according to the probabilities of pulmonary hypertension by CT and echocardiography showed no difference (Fig. 1). No significant association was found between bleeding risk and a moderate to high probability of pulmonary hypertension on echocardiography or a high probability of pulmonary hypertension on CT scan (Fig. 2).

To further assess the influence of the probability of pulmonary hypertension on postbronchoscopy bleeding, we adjusted for factors such as age and comorbidities and found no statistically significant differences between the candidate variables in either group. A proportional odds logistic regression model was adjusted, incorporating COPD, heart failure, sPAP (categorized as < 40, 40–60, and > 60 mmHg), and the number of biopsies taken during bronchoscopy as predictor variables. COPD was the only variable significantly associated with postbronchoscopy bleeding. Heart failure, sPAP and number of biopsies taken were not associated. No significant violations of the proportional odds assumption were found.

## Discussion

In this study, the prevalence of bleeding was 37.5%, and most cases were mild (76.9%). The frequency of bleeding was similar to that reported in studies performed in populations at sea level, which is not entirely unexpected, considering that bleeding in TBB does not originate from the pulmonary vascular bed. Only one patient presented severe bleeding, which is consistent with reported studies where severe bleeding events are extremely rare (0.031%)^4^. A large number of patients included had suspected pulmonary hypertension by CT scan of the chest (66.8%) and, less frequently, by echocardiography (31.2%), showing slight agreement between the two methods for assessing this probability. Despite the frequency of suspected pulmonary hypertension, the risk of bleeding was not significantly increased after TBB, regardless of whether the probability of pulmonary hypertension was assessed by echocardiographic or CT findings.

In previous studies, the prevalence of pulmonary hypertension in patients undergoing TBB was highly variable (1.2%−33.3%), possibly because the disease has been defined by indirect estimates^7,18^. The high prevalence in our group may be explained by a higher prevalence of pulmonary hypertension at high altitudes (defined as altitudes > 2500 m above sea level) or by sample selection bias, as the patients were included from a specialist center where high-risk patients who underwent TBB were referred. Although CT scans and echocardiographic findings are predictors of adverse clinical events in patients with pulmonary hypertension, they only estimate the probability of pulmonary hypertension but do not confirm its diagnosis^14^. Despite their limitations, the use of both tests could be useful in real life, since it is neither feasible nor necessary to perform direct hemodynamic measurements before performing endoscopic procedures^19^.

Pulmonary hypertension as a contraindication for bronchoscopy has been a debated topic for several years. Many pulmonologists consider this condition to be at least a relative contraindication^20^. Some studies have evaluated the safety of bronchoscopy in patients with pulmonary hypertension diagnosed by echocardiographic or CT findings. Takashima et al.^8^ reported, in a retrospective study of 314 patients who underwent TBB guided by radial endobronchial ultrasound (EBUS), that 11.1% of patients experienced bleeding. Of 17 patients with suspected pulmonary hypertension by echocardiography 5 (29.4%) bled and of 59 patients with suspected pulmonary hypertension by CT scan of the chest 11 (18.6%) bled, so the risk of bleeding was higher in patients with suspected pulmonary hypertension. A retrospective study of 190 patients who underwent TBB, EBUS-guided transbronchial needle aspiration, or endobronchial biopsy compared safety outcomes between patients with sPAP < 36 mmHg and those with sPAP ≥ 36 mmHg^12^. TBB was performed in 69 of 83 (83%) patients with low sPAP and 91 of 107 (85%) with high sPAP. Compared with 5 patients in the high-sPAP group, four patients in the low-sPAP group had minor bleeding (*p* = 1.00), with no difference in the incidence of major bleeding (1 patient in each group). Lashari et al.^11^, in a retrospective study of 394 patients who underwent TBB, compared complications between patients with and without pulmonary hypertension (mean pulmonary artery pressure ≥ 25 mmHg on right catheterization and right ventricular systolic pressure ≥ 40 mmHg on echocardiography), reporting no significant differences in the incidence of severe bleeding between the two groups (*p* = 0.491). However, prolonged intubation was more common in the group with pulmonary hypertension (*p* = 0.007). Ishiwata et al.^18^, in a retrospective evaluation of 145 patients who underwent diagnostic bronchoscopy at a single center in Japan, compared patients with and without clinical evidence of pulmonary hypertension (defined as a right ventricular systolic pressure ≥ 40 mmHg) reporting no differences in the incidence of postbronchoscopy bleeding between the two groups (18% vs. 16%; *p* = 0.742). In a systematic review of the literature with a total of 1699 patients, Ali et al.^17^ evaluated the presence of bleeding in patients with and without pulmonary hypertension who underwent TBB in six retrospective and three prospective studies^7,11,13,18,21–23^. The pooled OR for bleeding in patients with pulmonary hypertension was 1.01 (95% CI, 0.71–1.45). Similarly, in our study, we found no association between the risk of bleeding and the probability of pulmonary hypertension on echocardiography or CT scan. These findings may differ from those of other studies due to population selection and the small number of cases of moderate to severe bleeding; however, they further support that suspected pulmonary hypertension should not be a contraindication for performing bronchoscopy.

Our multivariate logistic regression model revealed that COPD was significantly associated with an increased likelihood of bleeding. Living at high altitude does not seem to influence the COPD prevalence^24,25^. COPD might increase the risk of bleeding due to airway inflammation in chronic bronchitis and elevated capillary pressure in patients with secondary pulmonary hypertension. Previously, Neuman et al. assessed the complications of TBB in 207 patients with COPD and reported a higher rate of hypoxemia and no association with postbronchoscopy bleeding in patients who had pulmonary hypertension diagnosed by echocardiography^22^. In a population-based study conducted in Florida State between 2000 and 2009 that estimated complications associated with TBB, COPD was associated with an increased risk for pneumothorax (OR 1.51, 95% CI 1.31–1.75) but not bleeding^26^.

This is one of the studies that has assessed the risk of bleeding of TBB with the largest number of patients included and, to our knowledge, is the first study performed in a high-altitude population. However, our findings should be interpreted considering certain limitations. First, a few cases of moderate or severe bleeding were observed, which may affect the reliability of the estimates. The low frequency of this complication in a single center would require a larger sample size to have sufficient events for analysis. There are several classifications of bleeding severity, which promote high heterogeneity in the reporting of this complication^27^; although none of them have proven to better predict outcomes, we used the recommended by the British Thoracic Society (BTS)^1^. Second, this is a retrospective study, which raises the possibility for information bias, since the accuracy of the data depends on their correct recording in medical records. Third, there could be selection bias, since patients with severe pulmonary hypertension are not routinely selected for bronchoscopy. Finally, the criteria used to define pulmonary hypertension are indirect measures, and we did not include the right catheterization for the diagnosis of pulmonary hypertension; this may reduce the internal validity of the study but increase the external validity, since it is a regular practice to make decisions based on echocardiographic and CT findings without a right catheterization prior to bronchoscopy.

In summary, in this high-altitude population, COPD predicted the likelihood of postbronchoscopy bleeding, whereas suspected pulmonary hypertension may not be a major determinant. This has implications for the risk stratification of patients undergoing TBB, who should not be ruled out solely on the basis of echocardiographic or CT probabilities of pulmonary hypertension. However, larger prospective studies are needed to confirm these findings and refine risk prediction models.

## Methods

### Study design and participants

A retrospective cohort study was conducted among patients who underwent bronchoscopy with TBB at a single tertiary care center in Bogotá, Colombia, from January 1, 2017, to October 10, 2024, and who had transthoracic echocardiography and CT scans of the chest performed three months before or after the date of bronchoscopy. Patients receiving outpatient care, with incomplete echocardiographic reports or poor image quality on CT scans were excluded.

Using the BTS criteria, we classified postbronchoscopy bleeding into no bleeding (traces of blood with no need for continuous suctioning, resulting in spontaneous cessation of bleeding), mild bleeding (use of continuous suctioning of blood from the airways, resulting in spontaneous cessation of bleeding), moderate bleeding (intubation of the biopsied segment with the bronchoscope in wedge position, use of adrenaline or cold saline solution to stop bleeding) or severe bleeding (bronchial blocker or catheter placement, application of fibrin sealant, or resuscitation, blood transfusion, intensive care unit admission or death)^1^. Once discharged, patients were considered no longer at risk.

### Data collection

For the present study, data from the database of bronchoscopies performed at our center were accessed in January 2025. Demographic data, clinical characteristics, underlying comorbidities, laboratory tests at admission (platelet count, prothrombin time and activated partial thromboplastin time), echocardiographic measurements, CT scans of the chest findings and outcomes were extracted from the hospital’s electronic medical records. Two researchers independently reviewed the records to double-check the data collected.

The echocardiographic probability of pulmonary hypertension was estimated with the maximum velocity of tricuspid regurgitation (m/s) and signs of pulmonary hypertension, according to the European Society of Cardiology guideline^14,28^. The estimated sPAP was also registered. The CT scan of the chest probability of pulmonary hypertension was defined as an increased pulmonary artery diameter (> 29 mm in men and > 27 mm in women) or the ratio of the pulmonary artery to aorta diameter (> 0.9), according to the recommendations of the Fleischner Society^29^. CT scans of the chest findings and pulmonary artery measurements were assessed according to the Fleischner Society glossary by a pulmonologist (JRL) with twenty years of experience^30^.

### Sample size calculation and sampling technique

The sample size was estimated using the rule of 10 events per independent variable for a multivariable model with six variables. Assuming an event proportion of 29.4%, a statistical power of 80% and a confidence level of 95%, a minimum of 204 participants was required^8^. Patients were enrolled consecutively until the target sample size was reached.

### Statistical analysis

Continuous data are presented as the means (standard deviations) or medians (interquartile ranges), depending on their distribution, whereas categorical variables are reported as frequencies and percentages. The data distribution was tested using the Shapiro-Wilk test. Between-group differences were assessed using a two-sided t test or the Mann-Whitney U test as appropriate.

Logistic regression analyses were performed to investigate the relationships between variables and postbronchoscopy bleeding. Predictor variables that were statistically significant were included in a multivariate logistic regression to determine those that could appropriately predict postbronchoscopy bleeding. The results of the regression analyses are presented in terms of estimated adjusted OR with corresponding 95% confidence intervals. Since bleeding severity is an ordinal variable, a proportional odds logistic regression model was implemented using the polr() function from the MASS package in R. The proportional odds assumption was evaluated using Brant’s test (brant.test() from the brant package) and by comparing the ordinal logistic model with a multinomial logistic regression model (multinom() from the nnet package) through a likelihood ratio test. A *p*-value < 0.05 in Brant’s test indicated a violation of the proportional odds assumption, in which case alternative models, such as multinomial logistic regression, were considered. Interaction terms were included to explore potential effect modifications, particularly between sPAP and chronic obstructive pulmonary disease (COPD), as well as heart failure, and the number of biopsies taken. Significant interactions were visualized using plots generated with the ggplot2 package.

Weighted Cohen’s kappa coefficient was applied to assess the agreement between echocardiographic and CT probabilities of pulmonary hypertension, using the interpretation criteria from Landis and Koch^31^.

Significance was determined at a two-sided *p-*value threshold of < 0.05. All the statistical analyses were performed using R studio version 2023.03.1.

## Data Availability

The datasets generated during and/or analyzed during the current study are available in the figshare database (https://dx.doi.org/10.6084/m9.figshare.29093561).
